# Task-specific fear rather than general kinesiophobia assessment is associated with kinematic differences in chronic low back pain during lumbar flexion: a preliminary investigation

**DOI:** 10.1097/PR9.0000000000001025

**Published:** 2022-08-05

**Authors:** Ryota Imai, Masakazu Imaoka, Hidetoshi Nakao, Mitsumasa Hida, Ren Fujii, Takehiro Shiba, Tomohiko Nishigami

**Affiliations:** a Graduate School of Rehabilitation, Osaka Kawasaki Rehabilitation University, Kaizuka, Japan; b Department of Rehabilitation, Medical Corporation Tanakakai, Musashigaoka Hospital, Kumamoto, Japan; c Industrial Measurement Co, Ltd, Osaka, Japan; d Department of Physical Therapy, Faculty of Health and Welfare, Prefectural University of Hiroshima, Hiroshima, Japan

**Keywords:** Fear of movement, Chronic pain, Kinesiophobia, Task-specific assessment, Low back pain

## Abstract

**Introduction:**

Kinematic data obtained during a movement task by individuals with chronic low back pain seem to be related to pain-related fear. General kinesiophobia assessments, such as Tampa Scale for Kinesiophobia, are often used to assess pain-related fear. However, these questionnaires could suffer from a lack of sensitivity and do not measure the fear of specific movements.

**Objectives:**

The purpose of this study was to investigate whether the task-specific assessment of pain-related fear exhibits a closer association with trunk kinematics during lumbar flexion compared with the general kinesiophobia in individuals with chronic low back pain.

**Methods:**

We assessed pain-related factors, task-specific fear, and Tampa Scale for Kinesiophobia-11 scores of 51 company employees. The lumbar angle during a lumbar flexion task was recorded by 2 wireless Axivity Ax3 accelerometers attached to the subject's spinous process (L3) and sacral spine (S2). Only task-specific fear was evaluated after the lumbar flexion task. We calculated the maximum lumbar flexion angle (°) and the peak angular velocity of lumbar flexion/return from flexion (°/s^2^). We conducted a hierarchical multiple linear regression analysis to determine variance explained in lumbar flexion task performance by task-specific fear after controlling for demographic, pain, and general kinesiophobia.

**Results:**

The results showed that task-specific fear was associated with the peak angular velocity of lumbar return from flexion (*R*^2^ adj. = 0.36, *P* < 0.01) and lumbar flexion (*R*^2^ adj. = 0.3, *P* = 0.01).

**Discussion:**

Our results suggest that clinicians should consider the potential added value of task-specific fear assessment over the sole use of conventional kinesiophobia assessment.

## Abstract 2

Clinicians should consider the potential added value of task-specific fear assessment over the sole use of general kinesiophobia assessment.

## 1. Introduction

Many people (40%–85% of most populations) develop low back pain (LBP) at least once in their lives.^11^ Patients with chronic LBP (CLBP) frequently present with impaired lumbar movements, such as limited movement velocity, hesitation of movement, atypical lumbar movement variability, and abnormal trunk muscle contraction.^18,23,24,26,32^ These motor behavioral changes may represent involuntary attempts to avoid pain by reducing spinal loading, but they may contribute to an increased risk of subsequent injury. Therefore, identifying the causes of these changes could be helpful in the treatment and prevention of CLBP.

Recent systematic reviews have reported no clear relationship between trunk kinematics and pain intensity in CLBP, and trunk kinematics were associated with pain-related fears and catastrophizing.^4,13^ The same has been reported for workers with CLBP, which is not indicative of definite tissue damage, and there is no clear relationship between the kinematics of the trunk and pain intensity of CLBP.^8^ Thus, these previous studies concluded that the factors involved in trunk kinematics are still unclear. In the “fear-avoidance model” of Vlaeyen and Linton,^33^ individuals undertake protective behaviors to avoid pain or to avoid movement(s) that might cause pain^19,21^; such behaviors could influence the development of the trunk kinematics in individuals with CLBP. Meulders et al. hypothesized that fear of movement-related pain influences trunk kinematics, although the movements themselves do not cause pain.^22^ Several studies have demonstrated that reduced speed of lumbar movement and hesitation of the movement were associated with the fear of movement as evaluated by self-reported questionnaires, such as the Tampa Scale for Kinesiophobia (TSK) and Pain Anxiety Symptoms Scale (PASS).^23,28,29^ However, such self-reported questionnaires could lack sensitivity because they do not measure the fear of specific movements or activities. The self-reported questionnaires administered before performing a particular movement may not reflect the “actual” fear during the movement because the questionnaires evaluate fear of general movement, not the target movement. The potential importance of task-specific fear has been highlighted in a review of the literature on the fear-avoidance model, which suggests the need to establish an assessment modality for the fear of specific movements.^24^

Recently, a preliminary study reported that specific-fear after a finger-tapping (FT) task in patients with a distal radius fracture could be evaluated by a visual analogue scale.^12^ In this FT task, a magnetic sensor was attached to the thumb and index finger of the patient to measure velocity and hesitation of movement. This task-specific assessment provided direct knowledge of the fear during a specific movement, rather than the more general fear of movement measured by general kinesiophobia. This study aimed to investigate whether the task-specific assessment of pain-related fear exhibits a closer association with trunk kinematics during lumbar flexion compared with the general kinesiophobia assessment in individuals with CLBP.

## 2. Subjects and methods

### 2.1. Design and subjects

This cross-sectional study was conducted at a company in Izumisano, Japan, between February 14 and 21, 2020. The recruitment period was from January 23, 2019, to February 13, 2019. Flyers that stated the inclusion and exclusion criteria were distributed to a company in Izumisano, Japan, and volunteers were recruited. The inclusion criteria were as follows: full-time employment at the company; the age of 20 to 65 years; LBP duration of >6 months; a score of 1 or more on an 11-point numerical rating scale (NRS; 0–10) for pain intensity; at least one previous visit to an orthopedic clinic due to LBP; and a diagnosis of LBP by the orthopedist. The exclusion criteria were as follows: part-time employment; previous spinal surgery; serious spinal pathology; pain at a site other than the lower back; acute lower back pain; psychiatric illness as diagnosed by a psychiatrist; or a diagnosis of neurological disease. Subjects with pain at a site other than the lower back and a diagnosis of neurological disease were excluded; therefore, we excluded subjects with sciatica (eg, lumbar pain radiating into the lower extremity). The study was intended to have 100 subjects, but 46 did not participate due to the influence of Covid-19; thus, a total 54 subjects (mean age, 47.2 years; range 25–63 years) fulfilled these criteria and participated in the study. In addition, we conducted a preliminary study because we are considering a large-scale “low back pain examination” and intervention for chronic low back pain patients who have fear of movement in the future.

This study was approved by Osaka Kawasaki Rehabilitation University (no. OKRU19-A012) and conformed to the 2008 Helsinki Declaration of Human Rights guidelines. Before the study started, written informed consent to participate was obtained from each subject. In addition, we report this study following the STROBE guidelines.^34^

### 2.2. Assessment questionnaires

First, subjects completed a questionnaire regarding their age, gender, weight, and height. Each subject's body mass index (BMI) was calculated by dividing the body weight (in kilogram) by height squared (in square millimeters).

Pain intensity (movement evoked pain) was assessed using an NRS scale (0 = no pain and 10 = the highest possible degree of pain) after the task.^23^ We chose pain intensity during movement because movement-evoked pain appears to be more relevant to understanding its relationship with kinematic analysis than usual pain intensity at rest.^14^ Task-specific fear was assessed by another NRS (0 = no fear and 10 = the highest possible degree of fear) after the task.^20^ Both pain intensity and specific-task fear were assessed at the end of each type of movement pace. Subjects were asked the following questions: “Please indicate the pain intensity you experienced during lumbar flexion on a scale of 0 (no pain) to 10 (the highest possible degree of pain),” and “Please indicate the fear of movement you experienced during lumbar flexion on a scale of 0 (no fear) to 10 (the highest possible degree of fear).”

The kinesiophobia of subjects was assessed using the TKS-11 Japanese version, which shows better internal reliability, identical construction, and known group validity compared with the 17‐item version.^15^ The TSK-11 is scored on a 4-point scale from 1 (strongly disagree) to 4 (strongly agree). Low back pain disability was assessed by a Japanese version of the Roland Morris Disability Questionnaire.^27^

### 2.3. Lumbar flexion task

For the lumbar flexion task, the subject stood upright in a natural posture and then bent forward at the start cue. As in previous studies,^28,29^ the subjects were asked to perform this lumbar flexion task at 2 paces—that is, at a speed that was comfortable for the subject and as fast as possible. In addition, the subjects were instructed to bend their lumbar spine forward until they had reached the maximum lumbar range of motion (ROM) and then to extend their lumbar spine backward and finally to return to an upright posture. The subjects practiced the tasks ahead of time so that they would not perform the task incorrectly and to check that the sensor attached to the subject would not come off. Each subject performed the lumbar movement task thrice, and a comfortable pace and the fastest possible pace were performed randomly. In addition, the subjects were allowed to take a 30-second break between the first and second task periods and between the second and third task periods. Among them, a 15-second rest was used for the kinematic analysis.

### 2.4. Kinematic data collection and processing

**Figure 1.**
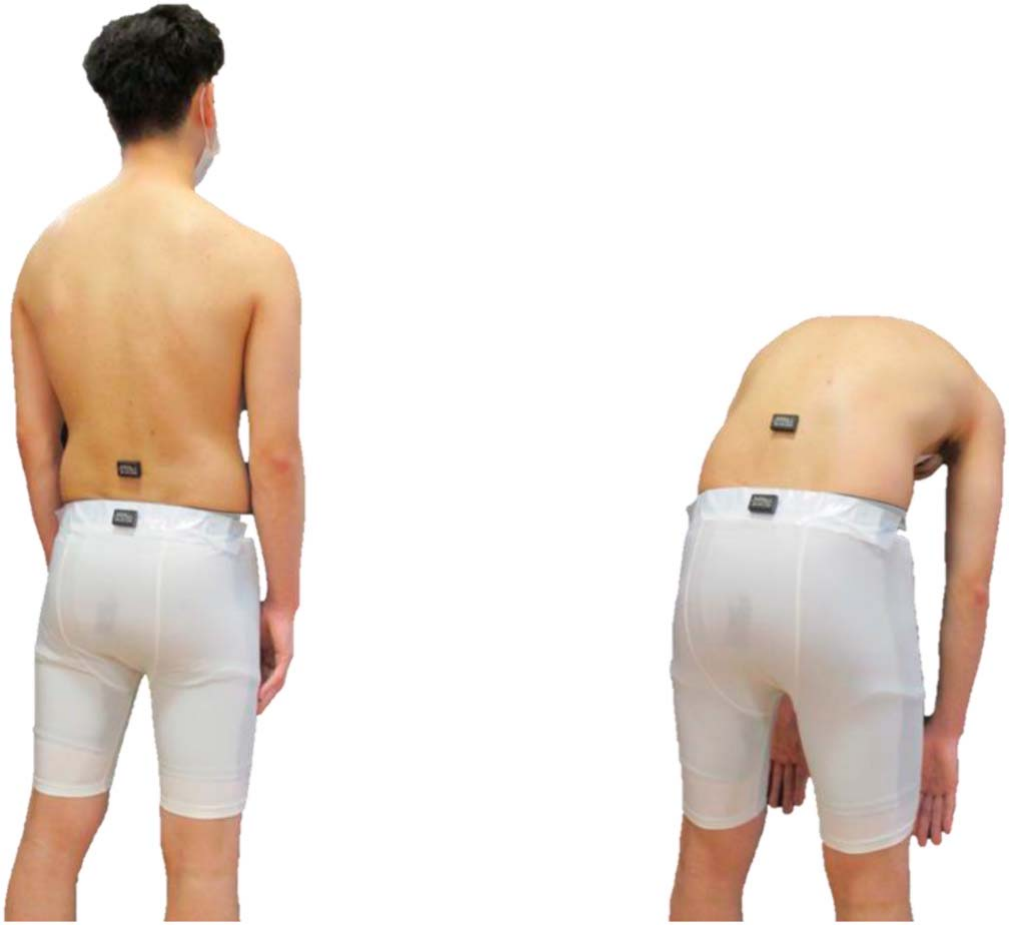
Bending task. The accelerometers were attached to the spinous process (L3) and sacral spine (S2). We recorded kinematic data during the lumbar flexion task.

We recorded the lumbar angle of the subjects during the lumbar flexion task with the use of 2 wireless Axivity Ax3 accelerometers (Axivity, York, United Kingdom). The Ax3 is a small (23 × 32.5 × 7.6 mm; 11 g), 3-axis accelerometer capable of logging acceleration data at 100 Hz. This device has been validated for recording human movement at high resolution.^5^ The 2 accelerometers were attached to the spinous process (L3) and sacral spine (S2) (Fig. 1), as described previously.^18^ The accelerometers were first calibrated to zero with the subject in a relaxed standing position, and the lumbar ROM was measured as the difference between the trunk angular displacement at L3 and pelvic angular displacement at S2.^18^ We calculated 3 parameters: (1) the maximum lumbar flexion angle for lumbar flexion (in degrees, °); (2) the peak angular velocity of lumbar flexion (°/s^2^); and (3) return from flexion (°/s^2^). The maximum lumbar flexion angle (°) and peak angular velocity of lumbar flexion (°/s^2^) were calculated in phase 1. The peak angular velocity of lumbar return from flexion (°/s^2^) was calculated in phase 2. We used the average of the peak angular velocity for each trial. In addition, phase 1 was from the start cue to the time point at which the subject's lumbar flexion angle was maximum. Phase 2 was from the time point at which the subject's lumbar flexion angle was the maximum to the time point at which the subject returned from flexion. Subjects were in a natural posture with a neutral lumbar spine position at both the time point at the start cue and the subject's return from flexion. Thus, the maximum lumbar flexion angle was the same for phases 1 and 2.

### 2.5. Statistical analyses

We compared kinematic data (the maximum lumbar flexion angle, peak angular velocity of lumbar flexion, and return from flexion) by performing a 1-way analysis of variance (ANOVA) and a post hoc test using Bonferroni correction.

We used Pearson correlation test to investigate whether the task-specific fear (NRS), and the TSK-11 results were related to the kinematic data. This correlation analysis was conducted to determine the objective variables and to investigate a correlation between task-specific fear and TSK-11. Because the significance level would change if the correlation analysis was repeated, Bonferroni correction was used to adjust the *P* values.^1^ In our study, a correlation analysis was conducted using 5 factors (age, BMI, the maximum lumbar flexion angle, the peak angular velocity of lumbar flexion, and return from flexion) for task-specific fear, TSK-11, and pain intensity. Therefore, the significance level of 0.05 was divided by 5, so the significance level was set at *P* = 0.01. In addition, Fisher z transformation was used to assess between-group differences of correlation coefficients.^9^ A z score outside the range of −1.96 ≤ z ≤ 1.96 indicated statistically significant differences between coefficients.

To investigate whether the task-specific assessment of pain-related fear exhibits a closer association with trunk kinematics during lumbar flexion than the general kinesiophobia in individuals with CLBP, we conducted a hierarchical multiple linear regression analysis using SPSS ver. 27.0 (IBM, Chicago, IL). Step 1 regression used the subjects' basic information (age and BMI), and step 2 used the same information plus the pain intensity and TSK-11 data. Step 3 adds task-specific fear. The significance level was set at *P* = 0.05. If there was no correlation between fear, age, and BMI, step 1 was deleted, and step 2 was carried forward. In this case, step 1 regression used pain intensity and TSK-11, whereas step 2 added task-specific fear. The variance inflation factor (VIF) was calculated to investigate multicollinearity. Multicollinearity was deemed present if the variance inflation factor was more than 5 because a VIF limit of 5 is stricter than a VIF limit of 10.^16^

## 3. Results

Four of the 54 subjects did not complete the evaluation. Thus, the final number of subjects for the analyses was 50 subjects. Tables 1 and 2 summarize the subjects' characteristics and the results of the clinical assessments and kinematic analysis.

**Table 1:** Subject characteristics and clinical information.

	Total (n = 54)	Min.	Max.
Age (y)	47.1 ± 11.1	25.0	63.0
Sex male, n (%)	29 (55.9%)		
BMI, (kg/m^2^)	22.3 ± 2.3	17.8	27.4
Duration (mo)	152.8 ± 129.6	6.0	480.0
Pain intensity (NRS), (0–10)	2.8 ± 1.9	1.0	9.0
Task-specific fear (NRS), (0–10)	2.9 ± 2.5	0	9.0
TSK-11, (11–44)	20.9 ± 5.4	11.0	32.0
RDQ, (0–24)	4.1 ± 4.1	0	19.0

Mean ± SD.

BMI, body mass index; NRS, numerical rating scale; RDQ, the Roland Morris Disability Questionnaire; TSK-11, Tampa Scale for Kinesiophobia.

**Table 2:** Subjects’ kinematic analysis at each trial.

		Average	Trial 1	Trial 2	Trial 3
Comfortable pace	Peak angular velocity of lumbar flexion (°/s^2^)	32.6 ± 12.9	32.6 ± 13.6	32.6 ± 13.7	32.7 ± 14.0
	Peak angular velocity of lumbar return from flexion (°/s^2^)	−31.9 ± 11.6	−31.9 ± 12.5	−32.0 ± 12.5	−31.9 ± 11.8
	Maximum lumbar flexion angle (°)	27.6 ± 10.5	27.6 ± 10.4	27.8 ± 10.3	27.5 ± 10.7
As fast as possible speed	Peak angular velocity of lumbar flexion (°/s^2^)	47.8 ± 16.8	48.0 ± 17.0	47.8 ± 16.5	47.7 ± 16.8
	Peak angular velocity of lumbar return from flexion (°/s^2^)	−47.6 ± 16.3	−47.7 ± 16.4	−46.7 ± 16.5	−47.6 ± 10.6
	Maximum lumbar flexion angle (°)	30.7 ± 9.0	31.0 ± 10.1	30.5 ± 11.0	30.7 ± 9.0

Mean ± SD.

### 3.1. The correlation analyses

**Figure 2.**
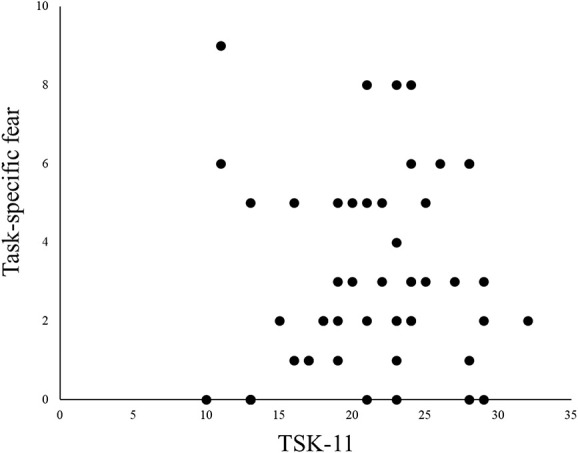
Result of correlation analysis of task-specific fear and TSK-11. The correlation between fear and pain was shown. TSK-11, Tampa Scale for Kinesiophobia.

The peak angular velocity of the lumbar region significantly correlated with task-specific fear (*r* = −0.43, *P* = 0.001) at a comfortable pace, and the peak angular velocity of lumbar return from flexion at a comfortable pace significantly correlated with task-specific fear (*r* = 0.41, *P* = 0.002) (Table 3). In addition, when performed as fast as possible, the peak angular velocity of the lumbar region significantly correlated with task-specific fear (*r* = −0.21, *P* = 0.009), and the peak angular velocity of lumbar return from flexion significantly correlated with task-specific fear (*r* = 0.59, *P* = 0.0004). Task-specific fear did not significantly correlate with the TSK-11 (*r* = 0.2, *P* = 0.16) (Fig. 2). When examining between-group differences in correlation coefficients, a significant difference in the peak angular velocity of lumbar return from flexion (as fast as possible speed) was found (Table 4).

**Table 3:** Pearson's correlation between kinematic data and fear assessment and pain intensity.

	Age (y)	BMI, (kg/m^2^)	Peak angular velocity of lumbar flexion (°/s^2^)	Peak angular velocity of lumbar return from flexion (°/s^2^)	Maximum lumbar flexion angle (°)
Comfortable pace					
Pain intensity (NRS), (0–10)	0.04 (0.778)	−0.03 (0.823)	−0.28 (0.08)	0.32 (0.02)	−0.02 (0.91)
Task-specific fear (NRS), (0–10)	−0.01 (0.955)	0.17 (0.253)	−0.43 (0.001)*	0.41 (0.002)*	−0.17 (0.54)
TSK-11, (11–44)	0.01 (0.962)	−0.06 (0.711)	−0.24 (0.08)	0.18 (0.19)	0.09 (0.5)
As fast as possible speed					
Pain intensity (NRS), (0–10)	0.04 (0.778)	−0.03 (0.823)	−0.17 (0.22)	0.13 (0.28)	−0.11 (0.31)
Task-specific fear (NRS), (0–10)	−0.01 (0.955)	0.17 (0.253)	−0.21 (0.009)*	0.59 (0.0004)*	−0.18 (0.25)
TSK-11, (11–44)	0.01 (0.962)	−0.06 (0.711)	−0.09 (0.52)	0.11 (0.42)	0.16 (0.29)

The *r* values denote the effect size of the correlation, and *P* values indicate significance. *r* (*P* value).

* Significant correlation (*P* < 0.01).

BMI, body mass index; NRS, numerical rating scale; TSK-11, Tampa Scale for Kinesiophobia.

**Table 4:** *z* score comparison.

	Comfortable pace		As fast as possible speed	
	Peak angular velocity of lumbar flexion (°/s^2^)	Peak angular velocity of lumbar return from flexion (°/s^2^)	Peak angular velocity of lumbar flexion (°/s^2^)	Peak angular velocity of lumbar return from flexion (°/s^2^)
Task-specific fear (NRS) vs TSK-11	−1.09 (1.72)	1.28 (0.2)	−0.62 (0.53)	2.86* (0.004)

*z* (*P* value).

* Significant correlation (*P* < 0.05).

NRS, numerical rating scale; TSK-11, Tampa Scale for Kinesiophobia.

### 3.2. Hierarchical multiple linear regression analyses

Step 1 was deleted, and step 2 was moved up because there was no correlation between fear, age, and BMI. In other words, step 1 regression used pain intensity, and TSK-11, step 2 added the task-specific fear. No significant differences were found between step 1 in any of the models. In step 2, this analysis showed that the peak angular velocity of lumbar flexion (comfortable pace) was associated with task-specific fear (*R*^2^ adj. = 0.29, *P* = 0.003), and the peak angular velocity of lumbar return from flexion (comfortable pace) was associated with task-specific fear (*R*^2^ adj. = 0.3, *P* = 0.006). In addition, we found that the peak angular velocity of lumbar flexion (fastest possible pace) was associated with task-specific fear (*R*^2^ adj. = 0.14, *P* = 0.02), and the peak angular velocity of lumbar return from flexion (fastest possible pace) was associated with task-specific fear (*R*^2^ adj. = 0.35, *P* = 0.0009) (Table 5). Because the VIF was less than 5, multicollinearity was determined not to be present.

**Table 5:** Hierarchical multiple linear regression analyses identifying the factors associated with kinematic data.

Factor	Comfortable pace							VIF
	Peak angular velocity of lumbar flexion (°/s^2^)							
	*R* ^2^	*R*^2^ adj.	*P*	β coefficient	*P*	95% CI		
Step 1	0.29	0.08	0.12					
Pain intensity (NRS), (0–10)				−0.19	0.19	−3.26	0.65	1.09
TSK-11, (11–44)				−0.17	0.24	−1.07	0.28	1.09
Step 2	0.5	0.29	<0.01					
Pain intensity (NRS), (0–10)				−0.13	0.33	−2.71	0.94	1.11
TSK-11, (11–44)				−0.14	0.29	−0.96	0.28	1.09
Task-specific fear (NRS), (0–10)				−0.4	<0.01	−3.44	−0.72	1.04

Factor	Comfortable pace							VIF
	Peak angular velocity of lumbar return from flexion (°/s^2^)							
	*R* ^2^	*R*^2^ adj.	*P*	β coefficient	*P*	95% CI		
Step 1	0.34	0.11	0.08					
Pain intensity (NRS), (0–10)				0.29	0.08	0.05	3.51	1.09
TSK-11, (11–44)				0.1	0.45	−0.37	0.83	1.09
Step 2	0.52	0.3	<0.01					
Pain intensity (NRS), (0–10)				0.23	0.1	−0.21	3.07	1.11
TSK-11, (11–44)				0.08	0.54	−0.39	0.73	1.09
Task-specific fear (NRS), (0–10)				0.37	<0.01	0.52	2.95	1.04

Factor	As fast as possible speed							VIF
	Peak angular velocity of lumbar flexion (°/s^2^)							
	*R* ^2^	*R*^2^ adj.	*P*	β coefficient	*P*	95% CI		
Step 1	0.19	0.04	0.46					
Pain intensity (NRS), (0–10)				−0.16	0.28	−4.29	1.29	1.12
TSK-11, (11–44)				−0.04	0.79	−1.01	0.83	1.12
Step 2	0.37	0.14	0.02					
Pain intensity (NRS), (0–10)				−0.09	0.54	−3.56	1.89	1.17
TSK-11, (11–44)				−0.02	0.9	−0.97	0.87	1.12
Task-specific fear (NRS), (0–10)				−0.59	0.02	−4.23	−0.34	1.06

Factor	As fast as possible speed							VIF
	Peak angular velocity of lumbar return from flexion (°/s^2^)							
	*R* ^2^	*R*^2^ adj.	*P*	β coefficient	*P*	95% CI		
Step 1	0.14	0.02	0.58					
Pain intensity (NRS), (0–10)				0.1	0.49	−1.67	3.39	1.09
TSK-11, (11–44)				0.09	0.57	−0.63	1.28	1.09
Step 2	0.6	0.35	<0.01					
Pain intensity (NRS), (0–10)				0.01	0.93	−2.02	2.19	1.11
TSK-11, (11–44)				0.04	0.78	−0.59	0.85	1.09
Task-specific fear (NRS), (0–10)				0.59	<0.01	2.31	5.44	1.04

NRS, numerical rating scale; TSK-11, Tampa Scale for Kinesiophobia; VIF, variance inflation factor.

## 4. Discussion

This study aimed to investigate which of 2 measures of pain-related fear—task-specific assessment or general kinesiophobia assessment—is more closely associated with trunk kinematics during lumbar flexion in individuals with CLBP. Compared with TSK-11, task-specific fear was significantly more related to the peak angular velocity of lumbar return from flexion (as fast as possible speed).

The major difference between general kinesiophobia assessment (eg, TSK) and task-specific fear is whether trait or situational kinesiophobia is being evaluated. Because general kinesiophobia assessment (eg, TSK) assesses beliefs related to the perceived harm and threat of experiencing LBP or performing physical activity while in pain, it cannot assess specific fears of movement. In addition, examples of specific activities (eg, trunk flexion and lifting weights) were not provided when patients responded to self-reported questionnaires, limiting their use in developing treatment programs. Conversely, we asked the subjects about the lumbar flexion task and assessed their fear after the task. Furthermore, because many patients have different fear behaviors, it may be better to use specific fears for an assessment to guide interventions. Thus, general kinesiophobia assessment (eg, TSK) involves a trait assessment, and task-specific fear involves a situation assessment.

Our results are consistent with those of previous studies.^3,35^ Campbell et al.^3^ showed that situational catastrophizing was more strongly associated with experimental pain responses than dispositional Pain Catastrophizing Scale scores in healthy subjects and arthritis patients. In addition, higher levels of situational catastrophizing are associated with lower pain thresholds and higher pain ratings. Woznowski-Vu et al.^35^ reported that situational catastrophizing significantly correlated with self-reported disability and pain questionnaires, both cross-sectionally and at a 3-month follow-up. This finding is very useful for clinicians who perform psychologically informed physical therapy interventions, such as cognitive functional therapy, which seeks to expose and modify what is experienced during a task (eg, task-specific fear) in physical therapy settings where the therapist can choose the assessment (task-specific fear NRS vs TSK-11). However, because our study was a preliminary investigation, the results should be interpreted with caution.

Our results did not show a significant correlation between kinesiophobia and maximum lumbar flexion angle, which is inconsistent with systematic reviews.^4,13^ Systematic reviews have shown an association between kinesiophobia and ROM.^4,13^ However, these systematic reviews did not include measures of angular velocity. A previous study showed that high pain-related fear was associated with angular velocity but not with joint angle.^30^ In addition, Fujii et al.^10^ reported that fear is associated with angular velocity. Recovery from an episode of low back pain follows a predictable course in which the recovery of normal lumbar angle is followed by recovery of normal peak angular velocity and finally angular acceleration.^30^ In the current study, the pain intensity and Roland Morris Disability Questionnaire scores were small, and the subjects were employed workers. Thus, because the subjects' pain was not intense nor was it interfering with their work, the angular velocity may have been affected without affecting the joint angle in our study. However, the practice trials completed before the lumbar movement task may have influenced the data.

Although other research groups have reported that the TSK was significantly correlated with kinematic data,^4,7,13,29^ our results are not consistent with those reports. Among patients with low back pain, it is unclear whether high pain intensity or kinesiophobia influences various movements or activities. To identify the movements or activities that are affected by pain or kinesiophobia, those movements or activities must be evaluated. Although some patients with CLBP may present with kinesiophobia of general movement, others might be fearful of specific activities only. Thus, CLBP patients who feel fear of specific activities may not show a high score on the general kinesiophobia, such as TSK or Fear Avoidance Belief Questionnaire. When asked about fear of general pain or fear of pain related to particular movements, patients will tend to imagine different contexts. Another reason may be that the self-reported questionnaires may lack sensitivity for measuring fear of a specific movement or activity. The potential importance of task-specific fear has been highlighted in a review of the literature on the fear-avoidance model, which suggests the need to establish a means of assessing fear of a specific movement.^24^

Our study has some limitations to consider. First, our results were obtained in a population with a moderate disability; therefore, they may not be applicable to all patients with CLBP. Compared with subjects of previous studies, the TSK-11 scores or pain intensity of our subjects were somewhat lower; therefore, all kinematic data might not be significantly correlated with the TSK-11 scores.^17,31^ Second, in the case of a longitudinal intervention, there is a need to assess fear before and after a movement or task so that fear-avoidance beliefs or expectations and the fear of the movement can be confirmed.^25^ In addition, kinesiophobia and pain factors related to the lumbar movement were not assessed after the practice trials. It is possible that failing to randomize subjects to different task orders and scale inquiries influenced the results in this study. It is possible that we were unable to create similar conditions in each trial using clear indicators. A previous study reported that the distinction between movement-evoked pain and resting or spontaneous pain is critically important.^6^ Our study did not distinguish between pain at rest and pain during a task or task-specific pain as possible confounding factors. Future research integrating sensory, psychological, and motor factors related to pain is required.^2,14^ Repeated full-range lumbar flexion may lead not only to the summation of pain but also to viscoelastic changes affecting the biomechanics of lumbar flexion movement through progressively reduced passive tissue resistance to movement. Third, adding a tool (eg, kinetic or electromyographic measurements) to allow further investigation of objective muscle activity without the interference of psychological factors would be of additional value. Our study was a preliminary study, and the sample size was relatively small. Thus, the results may differ if the sample size is increased and the experiment is repeated. Therefore, the results of this study should be interpreted considering that it is a preliminary study. Thus, we believe that this study can be improved by increasing the sample size and recruiting individuals with high kinesiophobia levels.

## 5. Conclusion

We sought to determine which of the 2 measures, task-specific fear (task-specific assessment) and general kinesiophobia (TSK), was more closely associated with trunk kinematics in individuals with CLBP. We found that, compared with TSK-11, task-specific fear was significantly more related to the peak angular velocity of lumbar return from flexion (as fast as possible). Our results suggest that clinicians should consider the potential added value of task-specific fear assessment over the sole use of conventional kinesiophobia assessments.

## Disclosures

The authors have no conflict of interest to declare.
